# Straight-to-Test Pathway in Faecal Immunochemical Testing (FIT)-Negative Patients: A Cost-Effective Approach

**DOI:** 10.7759/cureus.73464

**Published:** 2024-11-11

**Authors:** Amal Boulbadaoui, Muhammad Umair Rashid, Nandu Nair, Guleed Mohamed, Shawn Poh, Ingrid Britton, Ravivarma Balasubramaniam, Anne Gaunt, Philip Varghese

**Affiliations:** 1 Colorectal Surgery, University Hospitals of North Midlands (UHNM) NHS Trust, Stoke-on-Trent, GBR; 2 General Surgery, University Hospitals of Derby and Burton NHS Foundation Trust, Stoke-on-Trent, GBR; 3 Urology, University Hospitals of North Midlands (UHNM) NHS Trust, Stoke-on-Trent, GBR; 4 Radiology, University Hospitals of North Midlands (UHNM) NHS Trust, Stoke-on-Trent, GBR

**Keywords:** cancer, diagnostics, outpatient, pathway, triage

## Abstract

Introduction: Colorectal cancer (CRC) is the fourth most common malignancy in the UK and represents a high-volume diagnostic and clinical burden on the National Health Service (NHS). To maximise the use of limited diagnostic resources and increase efficiency, the colorectal services at University Hospitals North Midlands Trust (UHNM) developed the triage-to-test (TTT) service with risk stratification for diagnostic testing in patients with suspected colorectal cancer using faecal immunochemical testing (FIT) result. Our retrospective cohort study looked at the pick-up rate of colorectal cancer (CRC) and non-colorectal cancer (non-CRC) in FIT-negative patients.

Methods: The study was a retrospective review of all symptomatic patients over 18 years of age who had undergone FIT testing in the community between 1 November 2021 and 11 February 2022 and who were referred directly to the UHNM colorectal pathway from primary care (n=2,374). FIT negativity was set at <9.9 μg/g of faeces, as per the National Institute for Health and Care Excellence (NICE) DG30 guidelines. Patients were investigated and risk stratified in accordance with their FIT result and presenting symptoms.

Results: About 61.5% of patients referred were FIT negative (n=1,459) and 38.5% were FIT positive (n=915). Of those FIT-negative patients, 82 were excluded as their clinical outcomes were pending at the time of analysis. FIT positivity conferred a greater likelihood of colorectal cancer when compared with FIT-negative patients (p<0.0001). FIT-negative patients were most likely to have no significant pathology (32.5%, n=474). Incidence of colorectal cancer in the FIT-negative group was 0.5% (n=7) compared with 9.8% (n=89) in the FIT-positive group (odds ratio: 5.252, 95% CI: 4.012-6.875). Within the FIT-negative cohort, five patients were diagnosed with rectal cancer, one proximal descending colon cancer and one caecal cancer.

Conclusion : The use of a FIT-negative TTT pathway ensures that any symptomatic patients presenting with red flag symptoms can be investigated appropriately. It also provides reassurance to clinicians who have an ethical duty to investigate patients in whom they suspect sinister pathology. Moreover, a triage-to-Test pathway reduces outpatient capacity burden on healthcare trusts as they may send patients directly for investigation.

## Introduction

Colorectal cancer (CRC) is the fourth most common malignancy in the UK [1] and represents a high-volume diagnostic and clinical burden on the National Health Service (NHS). The classical ‘red flag’ symptoms (abdominal pain, rectal bleeding, weight loss and iron deficiency anaemia) have low positive predictive values (PPVs) for colorectal cancer [2]. Public Health England (PHE) introduced faecal immunochemical testing (FIT) in 2016 as a reliable quantitative assay to screen patients for colorectal cancer [3], with high sensitivity (93%) and specificity (91%) [4]. The use of FIT in primary care allows general practitioners to risk stratify patients presenting with lower gastrointestinal (GI) symptoms to decide which patients warrant referral to secondary care for further investigation. The National Institute for Health and Care Excellence (NICE) recommend a cut-off value of 10 µg/g [5] which can avoid unnecessary colonoscopy in 75%-80% of patients [6].

To maximise the use of limited diagnostic resources and increase efficiency, the colorectal services at University Hospitals of North Midlands (UHNM) NHS Trust developed the triage-to-test (TTT) service with risk stratification for diagnostic testing in patients with suspected colorectal cancer. Our retrospective cohort study looked at the pick-up rate of colorectal cancer (CRC) and non-colorectal cancer (non-CRC) in FIT-negative patients to discuss the economic and ethical justifications of investigating FIT-negative patients on our triage-to-test (TTT) pathway.

## Materials and methods

**Figure 1:**
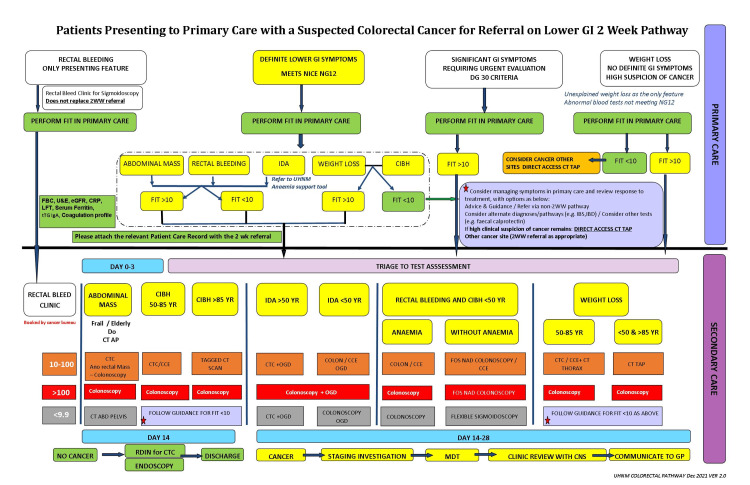
UHNM Colorectal Referral Pathway Algorithm University Hospitals North Midlands Trust (UHNM) NHS Trust Colorectal Referral Pathway created in 2021 as joint project by Colorectal Consultants and Administrative Staff. GI: gastrointestinal, FIT: faecal immunochemical testing, 2WW: 2 week-wait, NICE: National Institute for Health and Care Excellence, FBC: full blood count, U&E: urea and electrolytes, eGFR: estimated glomerular filtration rate, CRP: C-reactive protein, LFT: liver function test, IDA: iron deficiency anaemia, CIBH: change in bowel habit, CT TAP: CT thorax, abdomen and pelvis, IBS: irritable bowel syndrome, IBD: inflammatory bowel disease, CT AP: CT abdomen and pelvis, CTC: CT colonography, CCE: colon capsule endoscopy, OGD: oesophagogastroduodenoscopy, FOS NAD: flexible sigmoidoscopy (FOS) no abnormality detected (NAD), ABD: abdomen, RDIN: redirect investigation, MDT: multi-disciplinary team, CNS: clinical nurse specialist.

The study was a retrospective review of all symptomatic patients over 18 years of age who had undergone FIT testing in the community between 1 November 2021 and 11 February 2022 and who were referred directly to the UHNM colorectal pathway from primary care. FIT negativity was set at <9.9 μg/g of faeces, as per NICE DG30 guidelines [5]. Patients were investigated and risk stratified in accordance with their FIT result and presenting symptoms (Figure 1).

A total of 2,374 patients were triaged between November 2021 and February 2022 and all were included in the study. Statistical analysis was undertaken using SPSS software (IBM SPSS; Statistical Product and Service Solutions, Armonk, New York, United States) [7] using Chi-squared test, paired t-test and z score.

## Results

The median age in the FIT-negative group was 66 years vs 71 years in the FIT-positive group (Table 1). Patients were most likely to present in older age (modal age range at presentation for both FIT negative and FIT positive=71-80 years). There is a positive correlation between increasing age and FIT positivity observed: 32.58% (N=129) of patients under 50 years were FIT positive, increasing in the >80 age group (51.24%, N=206) (p<0.001). Older age is an independent risk factor for a higher FIT result along with neoplasia itself [8].

**Table 1:** Demographics of cohort (age, sex and FIT result) Count=N, i.e., number of patients. N%=percentage of overall patients. P-value=statistically significant when p<0.05. All p-square values exclusively calculated using Pearson's Chi-square test. FIT: faecal immunochemical testing. ** is the Chi-square value.

		Patient						Chi-square (P-value)
		FIT negative		FIT positive		Total		
		Count	Column N %	Count	Column N %	Count	Column N %	
Sex	Male	677	46.4%	427	46.7%	1,104	46.5%	0.016
	Female	782	53.6%	488	53.3%	1,270	53.5%	(p<0.900)
	Total	1,459	100.0%	915	100.0%	2,374	100.0%	
Age (years)	≤50Y	267	18.3%	129	14.1%	396	16.7%	
	51-60Y	258	17.7%	144	15.7%	402	16.9%	41.86**
	61-70Y	335	23.0%	165	18.0%	500	21.1%	(p<0.001)
	71-80Y	403	27.6%	271	29.6%	674	28.4%	
	81+ Y	196	13.4%	206	22.5%	402	16.9%	
	Total	1,459	100.0%	915	100.0%	2,374	100.0%	
FIT result (not large)	<9.9	1,459	100.0%	0	0.0%	1,459	61.5%	
	10-199.9	0	0.0%	691	75.5%	691	29.1%	2374.0**
	>200	0	0.0%	224	24.5%	224	9.4%	(p<0.001)
	Total	1,459	100.0%	915	100.0%	2,374	100.0%	

There were more female patients referred than male patients in both FIT-negative (female: 53.6%, N=782 vs male: 46.4%, N=677) and FIT-positive group (female: 53.3%, N=488 vs male: 46.7%, N=427) which reflects similar trends across the UK of a higher percentage of females than males presenting for screening [9]. Our study also demonstrated that despite more women being referred to our colorectal two week wait pathway, men were more likely to have a positive FIT result than women which is in keeping with current literature [10].

Pathology 

About 61.5% of patients referred were FIT negative (N=1,459) and 38.5% were FIT positive (n=915) (Table 2). Of those FIT-negative patients, 82 were excluded as their clinical outcomes were pending at the time of analysis. FIT positivity conferred a greater likelihood of colorectal cancer when compared with FIT-negative patients (p<0.0001). FIT-negative patients were most likely to have no significant pathology (32.5%, n=474). The most common bowel pathology detected in FIT-negative patients was diverticulosis (a benign condition characterised by small pockets or outpouchings of the colon that protrude through the muscular layer). Incidence of colorectal cancer in the FIT-negative group was 0.5% (n=7) compared with 9.8% (n=89) in the FIT-positive group (odds ratio: 5.252, 95% CI: 4.012-6.875). Within the FIT-negative cohort, five patients were diagnosed with rectal cancer, one proximal descending colon cancer and one caecal cancer. The incidence of anal cancer was similar in FIT-negative (0.2%, N=3) and FIT-positive groups (0.1%, N=1). Of the FIT-negative patients with colorectal cancer, four (57.1%) were found to have iron deficiency anaemia at index presentation. 

**Table 2:** All clinical outcomes recorded for this study Count=N, i.e., number of patients. N%=percentage of patients. P-value=statistically significant when p<0.05. All p-square values exclusively calculated using Pearson's Chi-square test. ** is the Chi-square value.

		Patient						Chi-square (P-value)
		FIT negative		FIT positive		Total		
		Count (N)	Column N (%)	Count (N)	Column N (%)	Count (N)	Column N (%)	
Outcome (pathology)	Adenoma	49	3.4%	204	22.3%	253	10.7%	
	Anal cancer	3	0.2%	1	0.1%	4	0.2%	
	Benign colorectal (other than adenoma)	170	11.7%	104	11.4%	274	11.5%	
	Benign non-colorectal	68	4.7%	93	10.2%	161	6.8%	
	Cancer (non-colorectal)	7	0.5%	15	1.6%	23	1.0%	540.32**
	Cancer (colon)	2	0.3%	54	5.9%	58	2.4%	(0.000)
	Cancer (rectal)	5	0.4%	36	3.9%	42	1.8%	
	Deceased	12	0.8%	6	0.7%	18	0.8%	
	Declined investigation	57	3.9%	20	2.2%	77	3.2%	
	Discharged	149	10.2%	33	3.6%	182	7.7%	
	Diverticulosis	289	19.8%	139	15.2%	428	18.0%	
	IBD	16	1.1%	25	2.7%	41	1.7%	
	None	475	32.4%	142	15.5%	615	25.9%	
	Not referred	5	0.3%	27	3.0%	32	1.3%	
	Other	70	4.8%	0	0.0%	70	2.9%	
	Pending	82	5.5%	16	1.7%	96	4.0%	
	Total	1,459	100.0%	915	100.0%	2374	100.0%	

**Figure 2:**
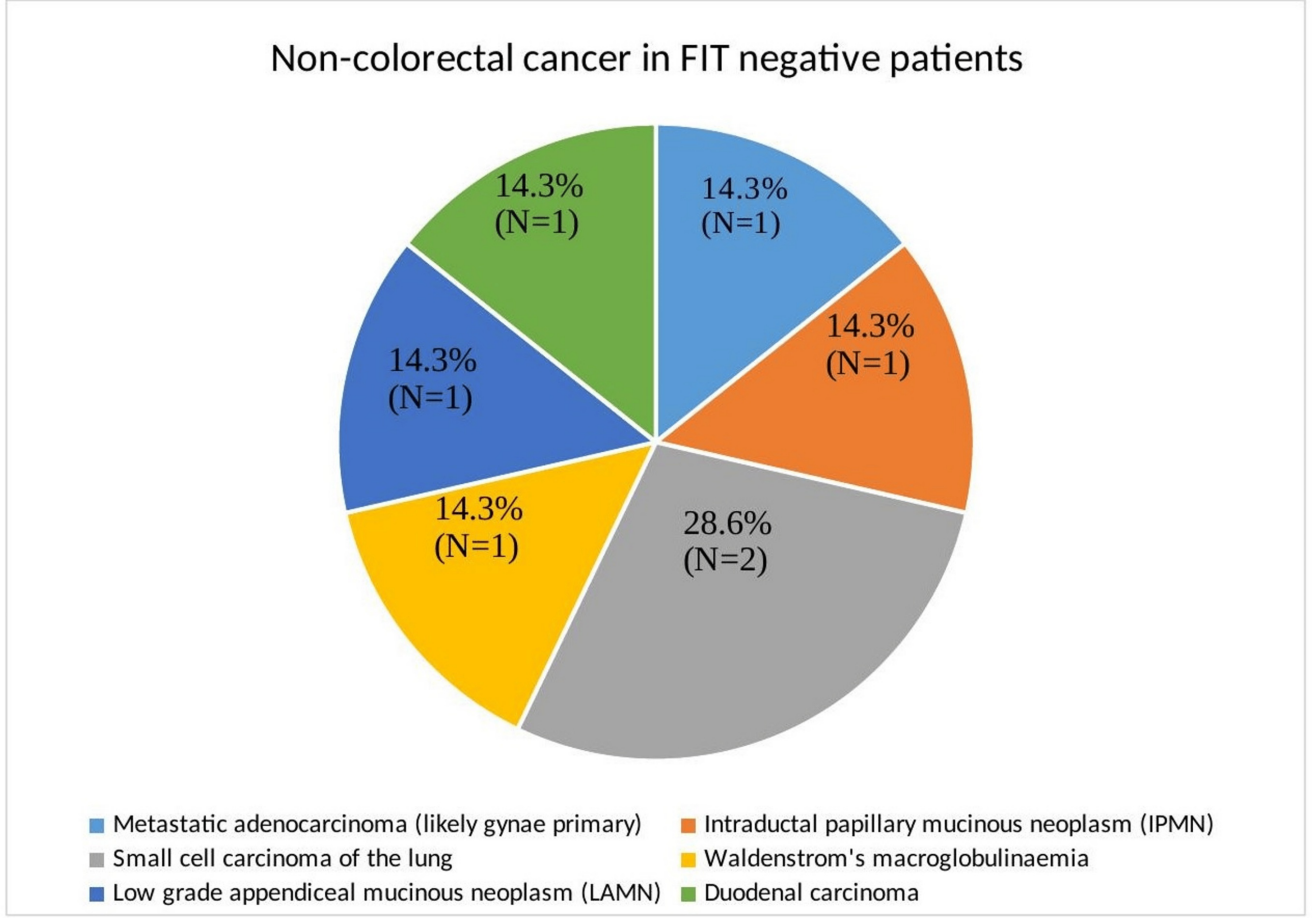
Non-colorectal cancer (non-CRC) in FIT-negative cohort (seven FIT-negative patients found to have non-CRC) FIT: faecal immunochemical testing, IBD: inflammatory bowel disease.

Non-colorectal cancer (non-CRC) was present in both groups: 0.5% of FIT-negative patients (N=7) had non-CRC and 1.6% (n=15) of FIT-positive patients were found to have non-CRC. The non-colorectal cancers found in FIT-negative patients is demonstrated below (Figure 2).

We also see that 19.4% (N=49) of adenomas within our patient data set were seen in FIT-negative patients, compared with 22.2% (N=204) in the FIT-positive cohort. All adenomatous polyps were excised at the time of colonoscopy. Of the 49 adenomas excised in the FIT-negative patients, 12.2% (N=6) were found to be size significant adenomas (defined as tubular adenomas ≥1 cm, or any adenomas with villous or high-grade dysplasia).

## Discussion

Our retrospective study supports the current literature evidence using FIT cut-off at <10 µg/g as a reliable initial triage test for CRC in primary care due to its high negative predictive value for colorectal cancer (9.8%, CI: 99.5%-99.9%). Despite this, it is not possible to conclusively exclude colorectal cancer or colorectal pathology on FIT results alone in patients with bowel symptoms. Our straight-to-test algorithm with a targeted CT abdomen and pelvis screen in symptomatic FIT-negative patients improved the lead time for diagnosis of CRC and non-CRC diagnosis (Figure 1). 

The need for greater engagement with the faster diagnostic standards (FDS) framework

Our triage-to-test (TTT) pathway allows for early detection of colorectal cancer and improved outcomes for patients. NHS England outlined its latest faster diagnostic standards (FDS) framework which includes the use of FIT in primary care as set out in guidelines by the Association of Coloproctology of Great Britain and Ireland (ACPGBI) [11]. The recommendation is for patients to be referred to secondary care despite a negative FIT if clinical concern remains [12,13]. The addition of FIT results has been useful in triaging referrals to the appropriate diagnostic test, thereby ensuring the use of the available colonoscopy capacity in the most efficient and economically justifiable manner. There remains a risk of both CRC and non-CRC in the FIT-negative cohort. Our study confirms a non-colorectal cancer rate in FIT-negative patients to be 0.5% which is comparable with the latest data of ~0.6% [14]. These cancers would likely have been picked up at a later stage were it not for engagement with the TTT service and availability of a CT abdomen and pelvis for screening of FIT-negative patients, and earlier detection of cancer translates to earlier treatment and better prognosis for patients. Furthermore, data shows that colorectal cancer-specific mortality in the UK ranks 10th in Europe for men and 14th for women [15]: the higher rate is likely due to patients presenting as acute emergency admissions [16]. This supports the argument for more accurate triaging in primary care for colorectal cancer to reduce the incidence of emergency cancer admissions.

COVID-19 and the growth of virtual triage consultations

COVID-19 revolutionised many aspects of NHS healthcare provision, and at UHNM Trust led to the development of our TTT pathway. This virtual clinic services bypasses face-to-face clinic and relies on symptoms and FIT test alone to order appropriate investigations. This is advantageous as it allows for streamlining of clinical and diagnostic services, reducing both outpatient clinic burden and cost [17].

If there was any clinical uncertainty about patient fitness for the straight-to-test pathway, patients were seen in face-to-face assessment clinic as an alternative. One review of orthopaedic virtual clinics found a 78.3% call back rate to face-to-face clinic [18]. However, in the context of orthopaedics it may be difficult to assess injury and healing; therefore, this should not be extrapolated to colorectal TTT clinics which remain a useful triaging pathway. Indeed, a straight-to-test pathway results in faster diagnosis and treatment as it bypasses the initial face-to-face assessment [19,20]. Our triage method resulted in significant reduction in outpatient burden capacity compared to the face-to-face service. It is at least non-inferior to fully face-to-face clinics. Unit time saved was another significant factor, freeing up capacity for other colorectal referrals.

A straight-to-test system also allows clinicians to pick up pathology that would have otherwise been missed. A recent meta-analysis found on average 23.6% of CT scans had incidental pathology that warranted further investigation or treatment [21], and our pathway allows symptomatic FIT-negative patients to be screened for potential non-colorectal pathology that may be causing their symptoms. This may also reduce GP referrals to other abdominal pathways (e.g. 2-week wait (2ww) urology/2ww gynaecology) after having had a negative CT.

Moreover, virtual clinics are being increasingly used in practice: the VOCAL mixed methods study looked at 300 hours of clinic data and found between 2% and 20% of all outpatient consultations were virtual by 2018, with the number rising after the COVID pandemic [22].

Economic and ethical considerations

FIT-negative patients referred onto our TTT pathway are most likely to be investigated with a CT scan if FIT negative or CT colonography if FIT negative with iron deficiency anaemia, both of which are cost-justifiable investigations. The cost of a diagnostic CT scan on the NHS stands at £154 [23]. Contrast CT has a 100% negative predictive value (NPV) for large CRC (95% CI: 95%-100%), which is similar to the NPV for all colorectal cancer and high-risk adenomas in CT colonography (98%) [24]. Although some studies have found the negative predictive value of cross-sectional imaging at 88.1% (95% CI: 82.8%-92.2%) [25], in the context of a FIT-negative triage-to-test pathway, a normal CT scan coupled with a negative FIT result provides greater reassurance to clinicians and patients alike.

Another consideration in straight-to-test pathways is the radiation exposure in FIT-negative patients and whether this is ethically justifiable. A standard CT abdomen and pelvis scan emits around 8-15 mSv [26]. If the higher dose of 15 mSv is used, this would be equivalent to 750 chest x-rays, giving a ~0.15% lifetime cancer risk [27] from this exposure. However, if CTs are clinically indicated but are not performed for fear of radiation, this may lead to incomplete or suboptimal studies, thereby delaying diagnosis or necessitating repeat examinations [28].

Furthermore, a straight-to-test pathway allows for reduced cost burden on services, not only in unit time but in financial terms, as fewer face-to-face outpatient appointments will be required (average cost per face-to-face clinic: £120) [29]. It also allows for greater efficiency, as there is an increasing number of did-not-attend (DNAs) in face-to-face clinics which equates to approximately 650,000 missed appointments monthly in the NHS [30].

Finally, resources in the NHS must be allocated in an ethical way to ensure patients in need of diagnostic investigations and treatments are not having these withheld or delayed. A straight-to-test pathway ensures greater diagnostic capacity as patients are triaged faster than if seen face-to-face initially [31-33]. Moreover, faster diagnosis reduces stress and concern from patients and for patients in whom TTT investigations do not show cancer, it may allow the referring clinician to move on to other potential causes for presentation [34]. Clinicians have an ethical duty to investigate symptomatic patients as well as provide them with the autonomy to make an informed choice, including risks of investigation [35]. Ethical duty also extends to ensuring the best outcome for patients, and our TTT pathway allows for patients to be investigated for cancer despite a negative FIT test [35]. The field of colorectal cancer testing is expanding too: NHS-Galleri [36] is a current randomised control trial that screens asymptomatic individuals to see if multi-cancer early detection (MCED) reduces late-stage cancer incidence. This is important in the field of colorectal cancer detection and raises further ethical discussions regarding cancer screening and may one day be used in conjunction with our FIT-negative pathway to assess patients more accurately.

Strengths and limitations of study

There were several strengths to this study: firstly, a large sample size of patients was obtained allowing for a greater validity of conclusions to be drawn, as well as reducing the margin of error. It also served as a service evaluation internally as we were able to identify the percentage of patients referred onto our pathway that had investigations complete and a diagnosis made within the faster diagnostic standards (FDS) framework. We found that the overwhelming majority of FIT-negative and FIT-positive patients met these standards. However, a limitation of the study was that this was a retrospective cohort study by design so other confounding factors that may have affected results could not be adjusted in real time.

## Conclusions

The use of a FIT-negative TTT pathway, which includes a CT abdomen and pelvis in colorectal cancer, ensures that any symptomatic patients presenting with red flag symptoms can be investigated appropriately to definitively rule out malignancy, despite a negative screening test. It provides reassurance to clinicians who have an ethical duty to investigate patients in whom they suspect sinister pathology, and not rely purely on statistical probabilities. Moreover, a triage-to-test pathway reduces outpatient capacity burden on healthcare trusts as they may send patients directly for investigation. Lastly, it remains clear that investigation of these FIT-negative patients can often result in important incidental findings that would not have otherwise been picked up, allowing for earlier treatment and management of pathology.
